# Innovative Semi-Nested Realtime PCR Assay with Extendable Blocking Probe for Enhanced Analysis of *SEPT9* Methylation in Colorectal Cancer

**DOI:** 10.3390/biomedicines12071458

**Published:** 2024-07-01

**Authors:** Linh Thuy Duong, Trang Thuy Dao, Hoai Thi Bui, Ung Dinh Nguyen, Ung Tien Hoang, Duc Viet Tran, Ba Van Nguyen, Tho Huu Ho

**Affiliations:** 1 Oncology Center, 103 Military Hospital, Vietnam Military Medical University, Hanoi 10000, Vietnam; bsduonglinh103@gmail.com (L.T.D.); tranduc170195@gmail.com (D.V.T.); bsnguyenvanba@yahoo.com (B.V.N.); 2 Department of Genomics and Cytogenetics, Institute of Biomedicine and Pharmacy (IBP), Vietnam Military Medical University, Hanoi 10000, Vietnam; daothuytrang_t63@hus.edu.vn (T.T.D.); buithihoai2704@gmail.com (H.T.B.); dr.ungd4.vmmu@gmail.com (U.D.N.); 3 Department of Rehabilitation, 103 Military Hospital, Vietnam Military Medical University, Hanoi 10000, Vietnam; bshoangtienungbvqy103@gmail.com

**Keywords:** colorectal cancer, *SEPT9* methylation, DNA melting, semi-nested PCR, extendable blocking probe, cell-free DNA, early cancer detection, cancer monitoring

## Abstract

(1) Background: The detection of methylated *SEPT9* (m*SEPT9*) in plasma is a promising approach to non-invasive colorectal cancer (CRC) screening. Traditional approaches have limitations in sensitivity and cost-effectiveness, particularly in resource-limited settings. (2) Methods: We developed a semi-nested realtime PCR assay utilizing extendable blocking probes (ExBP) to enhance the detection of low-level m*SEPT9* based on DNA melting. This assay allows for the discrimination of m*SEPT9* in the presence of high concentrations of non-methylated *SEPT9* (up to 100,000 times higher). (3) Results: The assay demonstrated a sensitivity of 73.91% and specificity of 80%, showcasing its ability to detect very low levels of methylated DNA effectively. The innovative use of ExBP without costly modified probes simplifies the assay setup and reduces the overall costs, enhancing its applicability in diverse clinical settings. (4) Conclusions: This novel assay significantly improves the detection of m*SEPT9*, offering a potential advance in CRC screening and monitoring. Its cost-efficiency and high sensitivity make it particularly suitable for the early detection and management of CRC, especially in settings with limited resources. Future studies are encouraged to validate this assay in larger populations to establish its clinical benefits and practical utility.

## 1. Introduction

Colorectal cancer (CRC) continues to be a major global health challenge, accounting for over 9.3% of all cancer-related deaths worldwide [1]. As the third most frequently diagnosed cancer and the second leading cause of cancer-related deaths, CRC resulted in approximately 1.9 million new cases and 0.9 million deaths globally in 2020 [2]. Despite advancements in detection and treatment, it is estimated that by 2030, the incidence of CRC will rise by 60%, leading to 2.2 million new cases and 1.1 million deaths annually [2]. Early detection remains crucial for improving survival rates, which can be as high as 90% at stage I but plummet to as low as 15% by stage IV [3].

Currently, approximately 25% of CRC cases are diagnosed at metastatic stages, underscoring the urgent need for effective early detection tools [4]. Although colonoscopy is the gold standard for CRC screening due to its high sensitivity and specificity [5], its invasiveness, high cost, and complexity limit its suitability as a universal screening tool. This has led to significant interest in developing non-invasive diagnostic tests that can enhance early detection and reduce unnecessary invasive procedures [6].

The first FDA-approved, blood-based test for CRC screening detects methylated DNA sequences of the *SEPT9* gene, highlighting its potential as a crucial biomarker for early CRC detection [7]. This test, known as the Epi proColon^®^ 2.0, has demonstrated its ability to detect CRC with a sensitivity of 80.6% and specificity of 96.9% [8]. However, its sensitivity for early-stage CRC (stages I and II) remains at 41.1% and 72%, respectively [9]. These figures suggest that while m*SEPT9* is a promising marker, its high sensitivity is more pronounced in stages III and IV rather than in the early stages. This indicates significant opportunities to enhance its detection accuracy, particularly for early-stage CRC, where intervention is most beneficial. Therefore, m*SEPT9* holds promise as a less invasive and more cost-effective diagnostic method. Enhancing its efficacy for early diagnosis could significantly improve outcomes and reduce the reliance on invasive procedures such as colonoscopy.

Enhancing sensitivity and specificity in CRC screening and early detection is crucial. This advancement not only relies on selecting or combining various methylation markers but also on developing advanced methodologies for analyzing methylated DNA. The Epi proColon^®^ 2.0 kit utilizes the HeavyMethyl method, which incorporates non-extendable blocking probes to enhance the selective amplification of methylated DNA while minimizing the amplification of unmethylated DNA. These non-extendable blocking probes aim to restrict the amplification of wild-type alleles (unmethylated DNA) by blocking potential priming sites on the wild-type template, which reduces mispriming. However, these probes often show limitations due to their binding inefficiency to the adenine and thymine-rich sequences typical of post-bisulfite converted unmethylated DNA, leading to suboptimal blocking and occasional non-specific amplifications.

In response, our recent development of extendable blocking probe (ExBP) technology has shown remarkable improvements over traditional non-extendable probes in preventing mispriming [10]. ExBP technology employs a novel approach that not only hybridizes to the wildtype sequence (e.g., post-bisulfite converted unmethylated DNA) to form a duplex but also initiates primer extension to form a more stable hybrid. This increased stability significantly enhances the assay’s selectivity by effectively blocking potential mispriming sites and reducing false amplification signals. Our research group has successfully demonstrated that this innovative method can prevent primer binding to wildtype alleles and detect mutant alleles, even in scenarios where wildtype alleles are present at concentrations 1000–10,000 times higher. This marked improvement in sensitivity and specificity underscores the potential of ExBP technology to transform CRC screening by detecting low-abundance methylated DNA with unprecedented accuracy.

By integrating the proven effectiveness of extendable blocking probes (ExBP) into the semi-nested realtime PCR assay, we aim to refine the analysis of *SEPT9* methylation (m*SEPT9*) in CRC. Unlike non-extendable blocking probes that require nucleotide modifications, ExBP functions as part of a standard primer without modification. This approach not only simplifies the assay design but also reduces costs, making it particularly suitable for settings with limited resources. Furthermore, the semi-nested PCR format enhances the sensitivity of the assay, enabling the detection of extremely low concentrations of tumor-derived cell-free DNA in the plasma. This combination of cost-efficiency and high sensitivity holds substantial promise for improving early CRC detection and management.

## 2. Materials and Methods

### 2.1. Study Participants

Participants were recruited from the 103 Military Hospital and the Vietnam National Cancer Hospital over a period spanning January 2021 to December 2023. The study was designed to collect blood plasma samples from a total of 96 individuals, aiming at a comprehensive evaluation of the novel semi-nested realtime PCR assay for the detection of circulating m*SEPT9*. This group included 46 CRC patients, stratified by disease stage to enhance understanding of the assay’s performance across the disease spectrum, with 36 patients at stages I to III and 10 at stage IV. The remaining 50 participants served as healthy controls, ensuring a balanced evaluation of the assay’s specificity and sensitivity.

Table 1 presents the demographic and clinical characteristics of the blood plasma sample providers, segregating data between cancer patients across various stages and healthy individuals. This structured approach allowed for an effective assessment of the assay’s efficacy in detecting m*SEPT9* at different stages of CRC and facilitated a comparative analysis with findings from healthy controls.

All participants were thoroughly informed about the research objectives and the nature of the study, and they voluntarily agreed to participate. This ensured a robust framework for the analysis of the results, supporting the validity and reliability of the study findings.

### 2.2. Sample Collection and Processing

Blood collection: Approximately 10 mL of venous blood was drawn from a different set of participants using K2 EDTA tubes to prevent clotting. The blood samples were processed within six hours to separate plasma, which was then stored at −80 °C.

DNA extraction and quality assessment: DNA was extracted from the plasma using the QIAamp Circulating Nucleic Acid Kit (Qiagen, Hilden, Germany). The quantity and quality of the extracted DNA from both sources were assessed using a Nanodrop spectrophotometer to ensure suitability for further analysis.

Bisulfite conversion: The extracted DNA underwent bisulfite treatment using the EZ DNA Methylation-Gold™ Kit (Zymo Research, Irvine, CA, USA). This treatment converts unmethylated cytosine to uracil (read as thymine during amplification) while leaving methylated cytosines unchanged. This step is crucial for the subsequent methylation-specific analyses.

### 2.3. Preparation of Standard DNA Samples

For the development and validation of our novel semi-nested realtime PCR assay, we prepared synthetic DNA calibrators to simulate bisulfite-converted sequences of the *SEPT9* gene. These calibrators were synthesized in two distinct forms: methylated and unmethylated versions of the *SEPT9* gene. The methylated DNA calibrators were synthesized in such a way that the cytosine residues at CpG sites were retained as cytosine, reflecting their methylated status. Conversely, the unmethylated DNA calibrators were also synthesized with cytosine at CpG sites but subsequently underwent bisulfite conversion, a process that converts all unmethylated cytosines to uracil. During PCR amplification, uracil is read as thymine, thus mimicking the chemical alterations that occur in bisulfite-treated DNA.

To accurately evaluate the assay’s sensitivity across a range of methylation levels, we prepared standard samples by mixing a constant concentration of unmethylated *SEPT9* DNA (10^6^ copies/µL) with varying concentrations of methylated *SEPT9* DNA. These mixtures were specifically designed to achieve methylated/unmethylated ratios of 10:1, 1:1, and extending to as low as 1:100,000. By establishing this gradient of methylation within the samples, we enabled precise quantification of the assay’s responsiveness to different levels of DNA methylation.

### 2.4. Semi-Nested Realtime PCR

The semi-nested realtime PCR assay designed for m*SEPT9* detection employs a two-round amplification process to ensure high specificity and sensitivity.

First-round amplification: In this round, the reaction mixture includes 1× HTOne MaX qPCR Green master mix (HT Biotec, Ho Chi Minh, Vietnam) and primers as detailed in Table 2. At the 5′ end, these primers include artificially engineered sequences (underlined) to enhance the amplification of AT-rich sequences. Simultaneously, their 3′ ends are specifically designed to anneal to non-CpG-containing sequences of the *SEPT9* gene, facilitating the co-amplification of both methylated and unmethylated DNA sequences. Amplification is initiated in an Applied Biosystems 9800 FAST Thermal Cycler (Thermo Fisher Scientific, Waltham, MA, USA) with a denaturation step at 95 °C for 15 min, followed by nine cycles of denaturation at 94 °C for 15 s, annealing at 50 °C for 60 s, and extension at 72 °C for 30 s. This is followed by 13 additional cycles with annealing and extension at 68 °C for 60 s. The amplified products are then diluted 20 times, and 5 µL of this solution is used for the second amplification round.

Second round amplification: The reaction mixture for the second round includes 1× HTOne MaX qPCR Green master mix and primers as detailed in Table 2. The primers are *SEPT9*/Fi with the ExBP sequence underlined to selectively enrich for methylated DNA and *SEPT9*_Ri. The reverse primer *SEPT9*_Ri used in the second round is identical to the sequence at the 5′ end of the primer *SEPT9*_Ro used in the first round, thus maintaining the semi-nested nature of the PCR. This round is performed using the Rotor-Gene Q instrument (Qiagen, Hilden, Germany) with an initial denaturation at 95 °C for 15 min, followed by one cycle at 94 °C for 15 s, 56 °C for 30 s, and 72 °C for 30 s, then 45 cycles at 94 °C for 15 s and 74 °C for 30 s. After the standard amplification cycles, a DNA melting curve analysis is performed to determine the melting temperature peaks of the PCR products at 78.0–78.7 degrees Celsius. Each sample is tested in triplicate, and a sample is deemed positive for m*SEPT9* if at least one of the three replicates shows a positive result, evidenced by the characteristic melting temperature peaks in the DNA melting curve analysis.

Quantification of total *SEPT9* levels: To ensure accurate interpretation of the methylation analysis, concurrent quantification of total *SEPT9* gene levels is executed alongside the methylation-specific testing. Total *SEPT9* gene levels are quantified in a parallel PCR reaction using the same reverse primer *SEPT9*_Ri and a specific forward primer (Table 2), following the thermocycling conditions of the second round.

### 2.5. Statistical Analysis

Statistical analyses in this study were performed using MedCalc software version 20.019 (MedCalc Software Ltd., Ostend, Belgium). For the evaluation of plasma samples from colorectal cancer (CRC) patients and healthy individuals, sensitivity, specificity, positive predictive value (PPV), and negative predictive value (NPV) were calculated based on the results of the semi-nested PCR assay. Confidence intervals (95% CI) were computed to provide estimates of the precision of sensitivity and specificity measures. To compare differences between groups, appropriate statistical tests, including the Mann–Whitney U test for non-parametric data and the Chi-square test for categorical variables, were applied.

To determine the diagnostic accuracy of the m*SEPT9* assay, receiver operating characteristic (ROC) curve analysis was conducted. The area under the ROC curve (AUC) was calculated to assess the overall effectiveness of the assay in discriminating between CRC patients and healthy controls. The optimal cut-off value for m*SEPT9* detection was determined using the Youden index from the ROC curve, maximizing the sum of sensitivity and specificity.

## 3. Results and Discussion

### 3.1. A Technical Overview of the Novel Semi-Nested Realtime PCR Assay for Detecting mSEPT9

**Figure 1:**
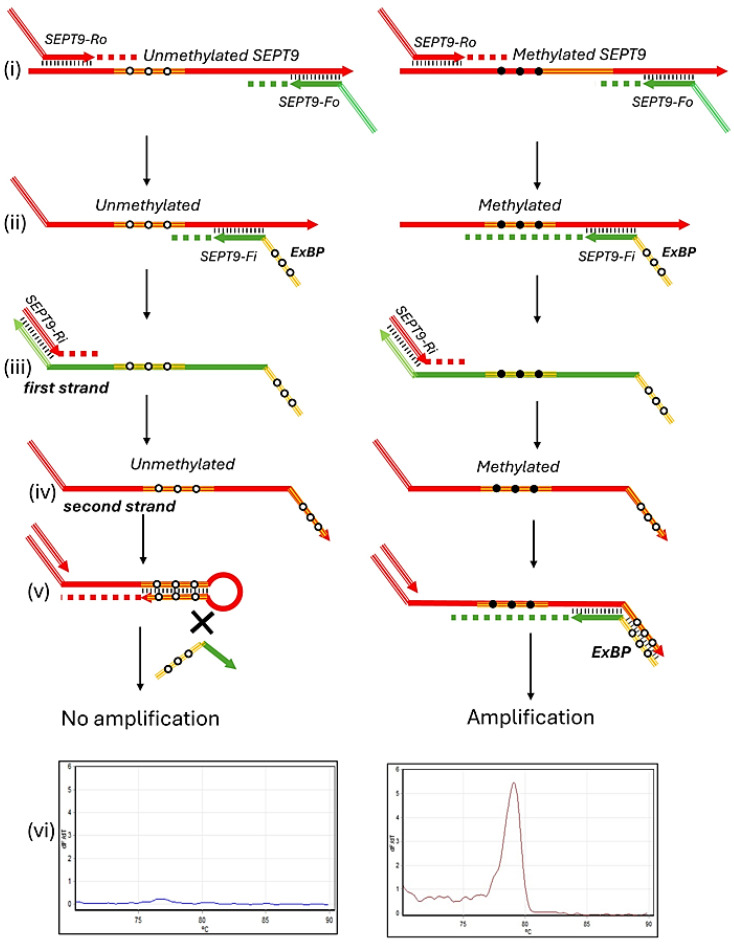
A schematic representation of semi-nested realtime PCR assay for the detection of m*SEPT9* based on DNA melting analysis. Figure 1 illustrates the semi-nested realtime PCR assay process, which is designed for high specificity and sensitivity in detecting m*SEPT9*. In Panel (**i**), the first round of PCR amplification is shown where forward and reverse primers, *SEPT9*_Fo and *SEPT9*_Ro, amplify both methylated and unmethylated sequences from bisulfite-converted DNA. These primers are specially designed to target regions devoid of CpG sites, allowing them to universally bind to DNA. Progressing to Panels (**ii**) through (**vi**), the second round of PCR is detailed. Panel (**ii**) introduces extendable blocking probes (ExBP) at the 5′ ends of the forward primer, which are designed to anneal to CpG-free regions, thus allowing its priming to both methylated and unmethylated sequences. The synthesis of the ‘first strand’, initiated by the forward primer incorporating the ExBP, is depicted in Panels (**ii**,**iii**). The reverse primer, *SEPT9*_Ri, used in the second PCR round is identical to the sequence at the 5′ end of *SEPT9*_Ro from the first round, maintaining the semi-nested nature of the PCR. This primer initiates extension on the first strand to form the ‘second strand’, as shown in Panels (**iii**,**iv**). Panel (**v**) highlights the formation of a stable hairpin structure at the 3′ end of the ‘second strand’—if derived from unmethylated DNA. This structure inhibits the strand from acting as a template in subsequent PCR cycles, thereby enhancing the selective amplification of methylated sequences. Finally, Panel (**vi**) describes the post-amplification phase where a DNA melting curve analysis is performed to determine the melting temperature peaks of the PCR products, verifying the specificity of the amplification and the methylation status of the DNA.

The semi-nested realtime PCR assay for m*SEPT9* employs a two-round amplification process to maximize specificity and sensitivity. The first PCR round amplifies both methylated and unmethylated *SEPT9* sequences, utilizing primers designed to anneal to regions devoid of CpG sites (Figure 1i). This approach allows for the co-amplification of all available *SEPT9* DNA, irrespective of its methylation status.

The forward and reverse primers, *SEPT9*_F and *SEPT9*_Ro, incorporate artificial sequences at the 5′ end that enhance amplification efficiency, particularly in AT-rich regions. These primers uniformly bind both methylated and unmethylated DNA, setting the stage for the subsequent discrimination between these forms in the second amplification round.

In the second PCR round, the integration of an extendable blocking probe (ExBP) at the 5′ end of the forward primer (*SEPT9*_Fi) plays a critical role in selectively amplifying methylated DNA sequences. The synthesis of the ‘first strand’ of DNA, initiated by this forward primer containing the ExBP, begins the process (Figure 1ii,iii).

The reverse primer (*SEPT9*_Ri) anneals to the ‘first strand’ and extends it to form the ‘second strand’ (Figure 1iii,iv). A key feature of this process occurs when the ‘second strand’ is derived from unmethylated DNA: a hairpin structure forms at the 3′ end of this strand. This hairpin structure, initially formed by the natural folding of the DNA sequence, is further stabilized by primer extension, effectively locking the strand and preventing it from acting as a template in subsequent PCR cycles (Figure 1v). This selective inhibition mechanism is vital, as it ensures that only methylated sequences are amplified, substantially enhancing the assay’s sensitivity and selectivity in detecting methylation.

In the post-amplification phase of the semi-nested realtime PCR assay, as detailed in Figure 1vi, a DNA melting curve analysis is conducted. This analysis is critical, as it identifies the melting temperature peaks of the PCR products, with the characteristic peak for methylated *SEPT9* sequences occurring at 78.0–78.7 degrees Celsius. This precise measurement not only verifies the specificity of the amplification but also confirms the methylation status of the DNA, ensuring the assay accurately reflects the sample’s methylation condition.

This semi-nested approach not only ensures the detection of methylated DNA in a background of unmethylated sequences but also improves the sensitivity of the assay by allowing a two-round amplification process. The broad amplification in the first PCR round is refined in the second PCR round to specifically target and amplify methylated sequences, thus improving the overall detection capability of low-abundance methylated DNA. This method is particularly advantageous for early cancer diagnostics and monitoring, where detecting low levels of methylation can be crucial.

The integration of ExBP within the standard PCR primer setup significantly simplifies the assay compared to traditional methods requiring complex and costly modified probes [11]. This simplification reduces the assay’s cost and complexity while minimizing the risk of non-specific amplifications, which are prevalent in highly sensitive PCR setups and can lead to false positives. Consequently, this semi-nested PCR assay offers a cost-effective, specific, and sensitive method for detecting m*SEPT9*, suitable for use even in resource-limited settings.

Further validation of this assay is detailed below, with a broader range of sample types being analyzed, including synthetic DNA standards and blood samples from both CRC patients and healthy controls. These studies aim to provide comprehensive data to confirm the assay’s practical applicability and diagnostic accuracy in a clinical setting, potentially enhancing diagnostic precision and contributing significantly to precision oncology by reliably detecting early methylation changes indicative of CRC.

### 3.2. Evaluation of the Novel Assay Using Standard Samples

To verify the innovative semi-nested PCR methodology for detecting m*SEPT9*, extensive evaluation was conducted using synthetic DNA standards. These standards simulated the post-bisulfite-modified *SEPT9* gene sequence and included both a fully methylated sample and a series of mixtures. Each mixture comprised a fixed concentration of unmethylated *SEPT9* DNA (10^5^ copies/µL) combined with varying concentrations of methylated *SEPT9* DNA to achieve methylated/unmethylated ratios of 10:1, 1:1, down to as low as 1:100,000.

**Figure 2:**
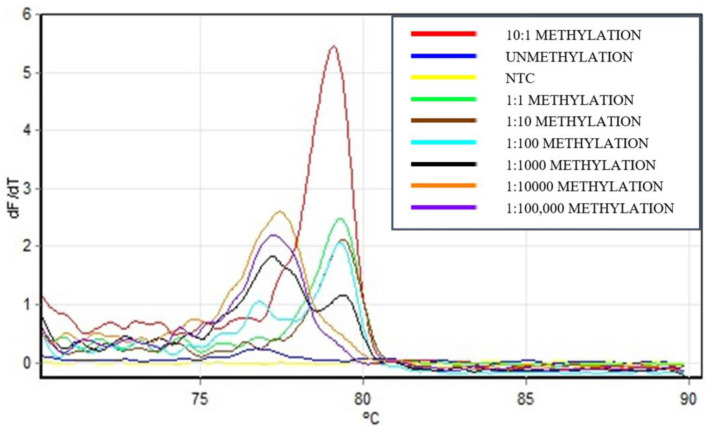
DNA melting curve analysis for m*SEPT9* detection. Representative melting curves are presented for a series of standard samples, each containing a constant concentration of unmethylated *SEPT9* DNA (10^5^ copies/µL) and varying concentrations of methylated DNA, resulting in methylated/unmethylated ratios from 10:1 down to 1:100,000, along with fully methylated (100%) and unmethylated (0%) controls. The characteristic melting temperature peak for methylated *SEPT9*, ranging between 78.0 to 78.7 degrees Celsius (highlighted in grey), is observed in the fully methylated sample and all spiked samples with ratios from 1:10,000 and higher. In contrast, this specific melting peak is absent in the unmethylated control and samples with less than 1:10,000 methylated to unmethylated ratio.

The results demonstrated that the fully methylated (100%) sample exhibited a distinct melting peak at 78.0–78.7 degrees Celsius, indicative of high methylation levels. This characteristic peak serves as a critical marker for confirming the presence of methylated DNA. Notably, the unmethylated (0%) sample showed no such peak, affirming the assay’s specificity. Moreover, mixed samples with ratios equal to or greater than 1:100,000 consistently exhibited a characteristic melting peak similar to that of the fully methylated standard (Figure 2). This observation confirms that the assay’s detection threshold is as low as 0.001%, highlighting its exceptional sensitivity and its capability to detect minimal levels of methylation.

The potential of our semi-nested PCR assay for precise detection of m*SEPT9* significantly enhances the early detection and monitoring of CRC. Our assay demonstrated the remarkable ability to identify m*SEPT9* levels as low as 0.001%, offering a hundredfold increase in sensitivity compared to the HeavyMethyl technique by Cottrell et al. (2004), which detected methylated DNA in a background of unmethylated DNA at a 0.1% detection limit [12]. Additionally, compared to a modified *SEPT9* assay reported to have a detection limit of 1:5000, equivalent to 0.02% methylated DNA in a background of unmethylated DNA [13], our method further extends this sensitivity to 0.001% or 1:100,000, highlighting the advanced capability of the semi-nested realtime PCR approach in detecting extremely low levels of methylation crucial for early cancer detection and monitoring.

This enhanced detection is largely attributed to the strategic use of extendable blocking probes (ExBP) in our assay, which selectively enriches methylated sequences by effectively blocking the amplification of unmethylated DNA. This specificity is critical in clinical settings, where precise quantification of methylation levels can significantly influence diagnostic and therapeutic decisions for CRC patients. This result lays a robust foundation for further applying this assay to clinical samples, aiming to validate its diagnostic utility extensively.

### 3.3. Evaluation of Plasma Samples from CRC Patients and Healthy Individuals

Following the validation of our semi-nested PCR assay on standard samples, we extended its evaluation to plasma samples from 46 patients with CRC and 50 healthy individuals. This part of the study aimed to assess the practical application of the assay in a real-world clinical setting, where biomarker levels in plasma samples are generally low, thus presenting a challenge for detection.

**Figure 3:**
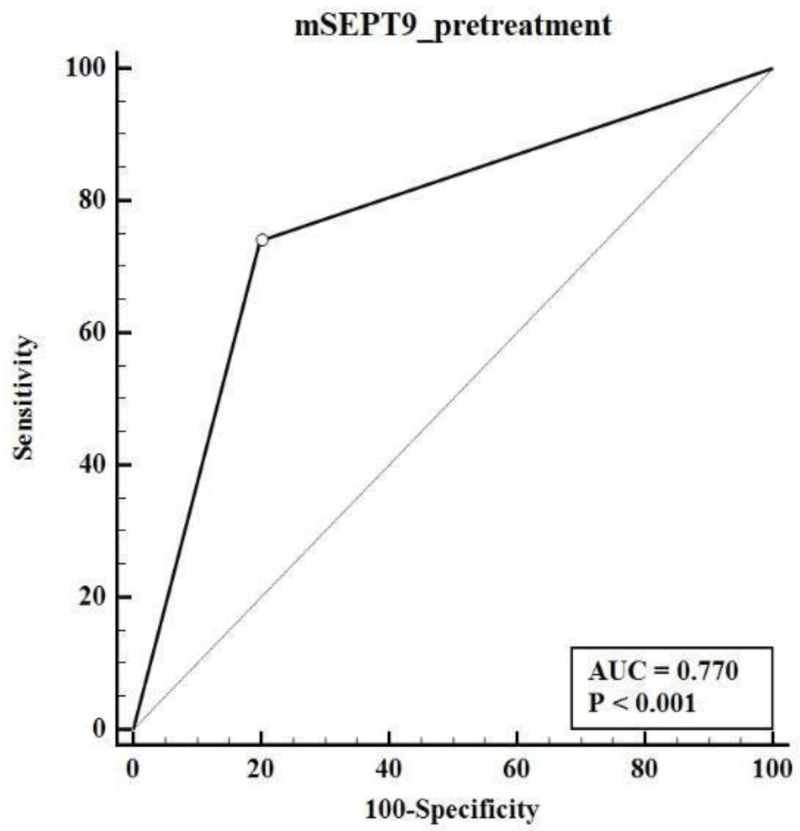
Receiver operating characteristic of m*SEPT9* in plasma. ROC curve displaying the diagnostic accuracy of m*SEPT9* status in plasma to differentiate between CRC and healthy individuals, with an AUC of 0.770.

Our findings revealed that 34 out of 46 CRC patients tested positive for m*SEPT9*, yielding a sensitivity rate of 73.91% with a confidence interval of 95% (CI: 58.87–85.73). Among the healthy controls, 10 out of 50 (20%) exhibited false-positive results, resulting in a specificity rate of 80% (CI: 66.28–89.97). The positive predictive value (PPV) and negative predictive value (NPV) of the assay were 77.23% and 76.92%, respectively, indicating the assay’s effectiveness in correctly identifying both true positive and true negative cases. The receiver operating characteristic (ROC) curve analysis further confirmed the assay’s moderate diagnostic accuracy with an area under the curve (AUC) of 0.77 (CI: 0.67–0.85) as shown in Figure 3.

The analysis of plasma m*SEPT9* in CRC patients revealed that its presence was not significantly correlated with several clinical and pathological factors such as gender, age, tumor location, T stage, N stage, M stage, tumor size, pathology of the tumor, or tumor differentiation, with all *p*-values exceeding the threshold for significance (Table 3).

Additionally, the presence of m*SEPT9* was not significantly associated with underlying morbid entities such as diabetes type 2 or other comorbid conditions, indicating no statistical correlation with these factors. However, a significant trend was observed in the positive rate of m*SEPT9* increasing with the disease stage, notably from 40% in stage I to 90% in stage IV, with stages III and IV exhibiting the highest positivity rates at 75% and 90%, respectively (Chi-square test, *p* = 0.049).

Comparing these results to those reported in the study by deVos et al. (2009) [13], our findings demonstrate a similar sensitivity but slightly lower specificity. The *SEPT9* methylation assay in deVos et al.’s study, which employed the HeavyMethyl technique, successfully identified 72% of cancers at a specificity of 93% in the training study and 68% of cancers at a specificity of 89% in the testing study. Notably, deVos et al. also observed an increase in the positivity rate with advancing disease stages, similar to the significant trend we observed in our study. Unlike the HeavyMethyl technique that relies on costly modified probes, our semi-nested realtime PCR assay utilizes standard primers without the need for expensive probe designs or modified nucleotides. This not only simplifies the assay setup but also potentially reduces costs, making the method more accessible for broader clinical applications. Furthermore, our study found no significant correlation between the presence of m*SEPT9* and underlying morbid entities such as diabetes type 2 or other comorbid conditions, suggesting that m*SEPT9* is a robust biomarker independent of these factors. The comparative analysis emphasizes the practical advantages of our innovative approach in enhancing the detection of *SEPT9* methylation for early cancer diagnosis and monitoring, aligning with the ongoing efforts to improve CRC screening efficiency and affordability in various healthcare settings.

### 3.4. Analysis of mSEPT9 before and after Surgical Treatment

This part of our study aimed to preliminarily evaluate the assay’s capability to monitor changes in the status of circulation m*SEPT9* before and after surgical treatment. In this focused analysis, among the CRC patients who tested positive for plasma m*SEPT9* prior to treatment, 16 had post-operative blood samples available for analysis one week following surgery (Table 4). Of these, 13 underwent surgeries with the intention of complete tumor resection. It was found that 10 of these patients showed a change from positive to negative circulating m*SEPT9* status, indicating successful tumor removal. In contrast, three patients remained positive for m*SEPT9*, pointing to potential residual disease. Specifically, one patient, who continued to test positive for plasma m*SEPT9*, had confirmed macroscopic residual tumor at the surgical margins, underscoring the assay’s capability to detect incomplete tumor resection. This highlights the value of m*SEPT9* as a biomarker for assessing surgical outcomes and monitoring for early signs of recurrence, offering critical insights for enhancing postoperative management in colorectal cancer care.

Further insights were gained from the analysis of metastatic CRC patients within this subgroup, reflecting on the study by Liu et al. (2021), which examined m*SEPT9*’s utility in monitoring response and predicting prognosis in stage IV CRC patients with liver metastasis [14]. Similar to their findings, our study observed that among five stage IV patients, two who underwent extensive surgeries including the removal of all visible tumors and associated metastases tested negative for m*SEPT9* post-operatively. These successful cases involved one patient with high rectal cancer and a single liver metastasis, who received concurrent resections of the high rectum and the left hepatic lobe, and another patient with sigmoid colon cancer that had invaded the left ovary and fallopian tube, who underwent complete excision of the left colon and reproductive structures, achieving clean margins (R0).

However, the remaining three patients, who only had their primary tumors removed and whose multiple liver metastases had not been addressed, exhibited varied post-operative m*SEPT9* results: two remained positive, indicating potential residual disease, while one unexpectedly tested negative. All three patients received the same maintenance therapy with bevacizumab and 5-FU-based chemotherapy regimens. The survival outcomes in these cases suggest a correlation, albeit tentative, given the small sample size: the two patients with continuous positive m*SEPT9* status post-surgery both passed away within 22 months post-operation. In contrast, the patient whose post-operative m*SEPT9* status turned negative remains alive and clinically stable under the same maintenance therapy, without signs of recurrence, 23 months post-surgery as of May 2024.

These findings, while provisional, highlight the possible role of m*SEPT9* as a marker for both residual disease and recurrence monitoring. Regular monitoring of m*SEPT9* in patients who initially revert to negative status post-surgery might help in early detection of recurrence, particularly if m*SEPT9* levels revert to positive. This could signal tumor regrowth, suggesting a need for more aggressive follow-up and treatment strategies. Given the limited number of cases, these observations are suggestive rather than definitive and underscore the need for further research with larger sample sizes to validate these potential correlations and enhance colorectal cancer patient management and outcomes.

## 4. Conclusions

Our study introduces a semi-nested realtime PCR assay that innovatively employs DNA melting curve analysis to detect circulating m*SEPT9* in colorectal cancer (CRC) patients. This new method leverages extendable blocking probes (ExBP), dramatically enhancing the detection of low-level methylated DNA, capable of discerning m*SEPT9* even in samples where non-methylated *SEPT9* is present at concentrations up to 100,000 times higher. It uniquely identifies m*SEPT9* by analyzing the characteristic melting temperatures of PCR products, a creative approach that enhances the assay’s capability to detect very low levels of methylated DNA amidst a high background of non-methylated *SEPT9*.

By avoiding the use of costly modified probes, our assay simplifies the setup and reduces costs, making it especially suitable for resource-limited settings. The technical sophistication of our assay, combined with its clinical performance—achieving a sensitivity of 73.91% and specificity of 80%—not only enhances its utility for CRC screening but also positions it as an invaluable tool for monitoring disease progression and recurrence. The observed correlation between a negative post-operative m*SEPT9* status and favorable patient outcomes further highlights its potential as a prognostic biomarker.

In line with the advantages of non-invasive methods highlighted by Ferro et al. (2021), integrating m*SEPT9* monitoring into clinical practice could provide a less invasive, cost-effective approach to managing colorectal cancer. Such methodologies align with the growing emphasis on liquid biopsies, which have been shown to offer significant benefits in terms of patient comfort, real-time monitoring, and economic feasibility [15].

These findings encourage the integration of our m*SEPT9* assay into routine clinical practice and set the stage for broader research. Future studies are urged to validate this assay in larger and more diverse populations, aiming to solidify its clinical applicability and confirm its benefits across different healthcare settings, thereby potentially revolutionizing CRC management through early detection and precise monitoring of treatment efficacy.

## Figures and Tables

**Table 1:** Demographic and clinical characteristics of plasma sample donors (*n* = 96).

Diagnosis	Colorectal Cancer (*n* = 46)	Healthy Controls (*n* = 50)
Sex		
Male	25	21
Female	21	29
Age (years)		
<55	15	32
55–70	26	18
≥70	5	0
Cancer stage		
I	5	-
II	7	-
III	24	-
IV	10	-

**Table 2:** Primer sequences for semi-nested realtime PCR.

Primers	Sequence	Amplification Round	Final Concentration
*SEPT9*_Fo	CGACGTAAAACGACGGCCAGT-CAACCCAACACCCACCT	First round	0.1 µM
*SEPT9*_Ro	CACACAGGAAACAGCTATGACCATG-GATTCGTTGTTTATTAGTTATTATGT		0.1 µM
*SEPT9*/Fi	TGGATTTTGTGGTTAATGT-AAATAATCCCATCCAACTA	Second round	0.1 µM
*SEPT9*_Ri	CACACAGGAAACAGCTATGACCATG		0.2 µM
Forward primer	AATCCGAAATAATCCCATCCAACTA	Quantification of total *SEPT9* levels	0.1 µM

**Note:** Underlined sequences in the primers *SEPT9*_Fo and *SEPT9*_Ro are artificially engineered sequences designed to enhance the amplification of AT-rich sequences. The primers *SEPT9*/Fi include the ExBP sequence underlined to selectively enrich for methylated DNA.

**Table 3:** Correlations between plasma m*SEPT9* and clinicopathologic characteristics of CRC patients.

Variables	N	m*SEPT9* (-) n (%)	m*SEPT9*(+) n (%)	*p*-Value
CRC	46	12 (26.08)	34 (73.91)	
Gender				
Female	21	6 (28.6)	15 (71.4)	0.73
Male	25	6 (24.0)	19 (76.0)	
Age				
<60	17	5 (29.4)	12 (70.6)	0.69
≥60	29	7 (24.1)	22 (75.9)	
Tumor location				
Colon	33	9 (27.3)	24 (72.7)	1.000
Rectum	13	3 (23.1)	10 (76.9)	
TNM Stage				
I	5	3 (60.0)	2 (40.0)	0.049
II	7	2 (28.6)	5 (71.4)	
III	24	6 (25.0)	18 (75.0)	
IV	10	1 (10.0)	9 (90.0)	
T stage				
T1	2	1 (50.0)	1 (50.0)	0.42
T2	8	2 (25.0)	6 (75.0)	
T3	16	5 (31.2)	11 (68.7)	
T4	20	4 (20.0)	16 (80.0)	
N stage				
N0	12	5 (41.7)	7 (58.3)	0.20
N1	29	5 (17.2)	24 (82.8)	
N2	5	2 (40.0)	3 (60.0)	
M stage				
M0	35	11 (31.4)	24 (68.6)	0.15
M1	11	1 (9.1)	10 (90.9)	
Tumor size				
<5	27	8 (29.6)	19 (70.4)	0.52
≥5	19	4 (21.1)	15 (78.9)	
Pathology tumor				
Carcinoma	40	10 (25.0)	30 (75.0)	0.64
Non-Carcinoma	6	2 (33.3)	4 (66.7)	
Tumor differentiation				
G1	0	0 (0.0)	0 (0.0)	
G2	38	12 (31.6)	26 (68.4)	0.089
G3	8	0 (0.0)	8 (100.0)	
General histological type				
Protrude	9	4 (44.4)	5 (55.6)	0.24
Ulcerative	22	6 (27.3)	16 (72.7)	
Others	15	2 (13.3)	13 (86.7)	
Comorbid disease				
No	29	10 (34.5)	19 (65.5)	0.09
Yes	17	2 (11.8)	15 (88.2)	

**Table 4:** Changes in plasma m*SEPT9* status before and after surgical treatment.

Patient ID	TNM Stage	Pre-Treatment m*SEPT9*	Post-Treatment m*SEPT9*	Surgical Treatment	Surgical Margin
01	T3N1aM0	Positive	Positive	Curative	R0
02	T4bN1cM0	Positive	Positive	Curative	R2
03	T2N1bM0	Positive	Negative	Curative	R0
04	T4aN1aM0	Positive	Negative	Curative	R0
05	T3N0M0	Positive	Negative	Curative	R0
06	T3N0M0	Positive	Negative	Curative	R0
07	T2N0M0	Positive	Negative	Curative	R0
08	T1N0M0	Positive	Positive	Curative	R0
09	T4aN1cM1c	Positive	Positive	Palliative	R0
10	T4bN1cM1	Positive	Positive	Palliative	R1
11	T4bN1cM1	Positive	Negative	Curative	R0
12	T3N1aM1a	Positive	Negative	Palliative	R0
13	T4aN2aM1a	Positive	Negative	Curative	R0
14	T2N2aM0	Positive	Negative	Curative	R0
15	T3N1bM0	Positive	Negative	Curative	R0
16	T3N1bM0	Positive	Negative	Curative	R0

R0: no cancer cells at the surgical margin; R1: microscopic cancer cells present at the surgical margin; and R2: macroscopic cancer cells visible at the surgical margin.

## Data Availability

The original contributions presented in the study are included in the article, further inquiries can be directed to the corresponding author.
